# Hospital Expenditure and Its Reduction

**Published:** 1905-01-21

**Authors:** 


### HOSPITAL EXPENDITURE AND ITS REDUCTION.

### PRINCIPLES IGNORED!

**LAST week we summarised a correspondence published in the *Daily Mail* on the subject of hospital expenditure and its reduction. Mr. Sydney Holland has ignored the principles raised by confining himself almost entirely to one matter of detail, which he must know cannot be thrashed out in the early stages of the discussion. His methods as a controversialist are sufficiently revealed by the following extracts from the correspondence, which speak for themselves:—**

SIR HENRY BURDETT.

December 24th, 1904.

"Every member of the medical profession attached to a hospital should be paid for the medical service he renders. At a rough estimate it would cost the London hospitals £60,000 a year to pay the doctors—or, say, about £6 10s. per occupied bed."

MR. SYDNEY HOLLAND.

December 27th, 1904.

"The sum Sir Henry suggests is £6 10s. per occupied bed. This would work out at under £150 per bed to each member of the staff—a magnificent sum indeed with which to remunerate a man earning his thousands a year for visiting his hospital three times a week!" "If the services of these men are to be adequately remunerated in money, the sum suggested by Sir Henry Burdett is absurdly inadequate."

December 31, 1904.

"I estimate that the majority of the staff at a great hospital would receive £300 a year each, a sum not absurdly inadequate. Substantial remuneration should be provided for about £60,000 a year. How many doctors are earning 'thousands a year' in London?"

January 4, 1905.

"Sir Henry Burdett *repeats his estimate that £6 10s. per occupied bed would be sufficient to pay the medical staff of a hospital an adequate salary*—a salary 'substantial' enough, he calls it, to reduce them from the position of honorary officers into that of paid officials. I replied this would work out at the magnificent sum of £150 a year, and *he replies that it would work out at £300 a year*, which he considers substantial. Take the London. There were 676 occupied beds in 1903. The sum to be divided among the 34 members of the staff would be therefore £4,384, equals £129 each. So to carry out Sir Henry's suggestion of £300 a year, we shall need at once to increase our expenditure not by £4,384 but by £10,200."

| January 14th, 1905. | January 18th, 1905. |
| --- | --- |
| "I did not repeat my estimate of £6 10s. per occupied bed, as Mr. Holland states. That estimate was but a rough and obvious calculation based on the total number of beds divided into the estimated cost, £60,000, for medical service. This calculation bore no relation to the apportionment of the total sum among the hospitals or the members of each staff. | "*Sir Henry alters his estimate, and in his last letter blames me for not noticing that he did not stick to his original one.*" |

If and when it is decided to abolish free medical service at the voluntary hospitals, it will be necessary to discuss in detail the rate of emolument which each member of the staff shall be paid for the services he renders. The amount of work done, the hours given to it, the nature of the work, the position and services of each member of the staff, and the circumstances of each hospital in regard to medical education, must all be taken into account to arrive at a basis of remuneration for the medical service rendered at each hospital by each physician and surgeon. A great hospital may be defined as one to which a medical school is attached, where the medical work is necessarily heavier than at an ordinary hospital. It is sufficient at this stage of the controversy to roughly estimate that individual payments should range from a maximum of £300 a year downwards. Having regard to the fees paid for a consultation in a private house, and to the fact that every member of a hospital staff gets very great advantages from his connection with the hospital, a rough estimate of this kind may properly be held at the outset to offer a sufficient basis for discussion.

